# Foot Disease Management by General Practitioners in People With and Without Diabetes: An Analysis of Nationally Representative Primary Care Data in Australia

**DOI:** 10.1002/jfa2.70066

**Published:** 2025-08-28

**Authors:** Peter A. Lazzarini, Hylton B. Menz, Cylie M. Williams, Julie Gordon, Susanna Cramb, Christopher Harrison

**Affiliations:** ^1^ Australian Centre for Health Services Innovation and Centre for Healthcare Translation School of Public Health and Social Work Queensland University of Technology Brisbane Australia; ^2^ Allied Health Research Collaborative The Prince Charles Hospital Brisbane Australia; ^3^ Discipline of Podiatry School of Allied Health, Human Services and Sport La Trobe University Melbourne Australia; ^4^ School of Primary and Allied Health Care Monash University Frankston Australia; ^5^ WHO‐CC for Strengthening Rehabilitation Capacity in Health Systems School of Health Sciences Faculty of Medicine and Health University of Sydney Sydney Australia; ^6^ Metro North Health Jamieson Trauma Institute Brisbane Australia; ^7^ Menzies Centre for Health Policy and Economics School of Public Health Faculty of Medicine and Health University of Sydney Sydney Australia

**Keywords:** diabetic foot, foot disease, foot ulcers, general practice, primary care

## Abstract

**Introduction:**

Foot disease is a leading cause of national disease burdens and is driven by diabetes. General practitioners (GPs) play a gatekeeper role in many national healthcare systems. Yet, national foot disease management by GPs has not been explored. We explored the management of foot disease by Australian GPs in people with and without diabetes.

**Methods:**

We analysed 16 years of annual, cross‐sectional, GP encounter data from the nationally representative Australian Bettering the Evaluation and Care of Health study in which a foot disease problem was managed. Factors independently associated with foot disease encounters by GPs were assessed using multivariable logistic regression.

**Results:**

Foot disease management rates increased from 11.6 per 1000 GP encounters (95% CI: 10.8–12.5) in 2000–2001 to 14.4 (13.3–15.4) in 2015–2016 and 6.1 (5.7–6.6) to 8.7 (8.1–9.3) per 100 Australian people. The rate of GP foot disease management was 2.4‐fold higher in people with diabetes compared to those without diabetes (31.5 [26.4–36.6] vs. 12.9 [11.8–14.0]). Foot disease encounters were positively associated with diabetes, male patients, older patients, English‐speaking backgrounds and having healthcare concession cards (all, *p* < 0.05); for patients with diabetes, only males were positively associated. Most frequent management actions used were medications, procedures and pathology with referrals, counselling and imaging least frequent.

**Conclusions:**

Australian GP management rates for foot disease are higher than many more well‐known health conditions and increasing. GPs frequently manage foot disease with medications and procedures, but relatively rarely counsel or refer. Future strategies to improve GP foot disease management and referrals are needed.

## 1 Introduction

Foot disease is a leading cause of the total global burden of disease [1, 2]. It is defined as peripheral neuropathy, peripheral artery disease, ulceration, infection or amputation affecting the foot [2, 3]. Diabetes‐related foot disease causes around 60% of all amputations and 2% of all hospitalisations globally [1, 2, 4]. Whereas nondiabetes‐related foot disease causes around 30% of amputations and 2% of all hospitalisations [5, 6]. Major amputation rates (above ankle) caused by foot disease are decreasing worldwide [4, 7]; however, hospitalisation and minor amputation rates (below ankle) caused by foot disease are increasing [4, 7]. Implementing diabetes‐related foot disease management that adheres to guideline recommendations has been found to reduce hospitalisations and amputations [8, 9]; however, adherence to these guidelines in practice is low [9, 10]. Although studies have explored foot disease management in nationally representative secondary and tertiary care services [9, 10], none to our knowledge have done so in primary care [11].

Studies of nationally representative primary care systems in the United Kingdom and Australia have found around 3 per 100 general practitioner (GP) consultations manage some form of foot‐related conditions [11, 12, 13, 14], and all were associated with older age and females [11, 12, 13, 14]. Surveys of GPs have also found GPs rarely refer and typically only manage foot‐related conditions when patients complain [15, 16]. Whereas other studies report that most people hospitalised for diabetes‐related foot disease have never been referred to a multi‐disciplinary footcare team [4, 16]. With GPs as a gatekeeper for many national healthcare systems, exploring GP management of health conditions in nationally representative primary care services is critical to informing national policy and practice [11, 17]. Therefore, this study aimed to explore the rate at which foot disease is managed by GPs in Australia, the factors associated with these foot disease consultations and the actions taken by GPs to manage foot disease in people with and without diabetes during these consultations.

## 2 Methods

### 2.1 Study Design

This study analysed data between 2000 and 2016 from the Bettering the Evaluation and Care of Health (BEACH) study, an annual cross‐sectional study of nationally representative GP activity in Australian general practice. BEACH data have been used to inform real‐world GP and primary care practice in over 300 peer‐reviewed articles [18]. Ethical approval for the BEACH study was obtained from the Human Research Ethics Committee of the University of Sydney (Ref: 2012/130) and the Australian Institute of Health and Welfare Ethics Committee (1998–2011). The methodologies employed in BEACH have been described in detail elsewhere and are summarised below [18]. This study is reported according to the Strengthening the Reporting of Observational studies in Epidemiology statement (Supporting Information S1: Appendix Table A1).

### 2.2 Study Population and Settings

Each year of the study period, a new random sample of 1000 GPs across Australia captured a range of variables from 100 consecutive patient encounters between April and March [11, 18]. Encounters were defined as either direct or indirect consultations between a GP and patient, with ∼99% being direct consultations [18]. This resulted in approximately 100,000 GP‐patient encounter samples captured each year and is highly representative of all GP activity in Australian general practice [11, 18].

### 2.3 Variables Collected

All participating GPs completed a standardised profile form capturing variables on their age, sex, practice postcode, country of graduation and years of experience [18]. Each GP then completed a structured patient encounter form to capture variables on each consecutive patient encounter with a consenting patient in the following domains: demographics, patient reason(s) for encounter, problems/diagnoses managed and management actions [18]. Patient demographics included age, sex, residential postcode, Aboriginal and/or Torres Strait Islander (respectfully referred to as Indigenous peoples), non‐English speaking background and healthcare concession card status. Postcodes were used to define the practice's location and patient socio‐economic level [17, 18]. Up to three patient reasons(s) for the encounter and up to four problems managed were recorded at each encounter. Management actions for each problem managed were also recorded. All details recorded by the GP were entered into a database by trained clinical coders [18]. Patient reasons for encounters, problems managed and any nonpharmaceutical management actions were coded using ICPC–2 PLUS, which automatically classifies data to the International Classification of Primary Care, second edition (ICPC–2) [18, 19]. Pharmaceutical management actions were coded using the Coding Atlas of Pharmaceutical Substances and mapped to the World Health Organisation's Anatomical Therapeutic Chemical Classification System [18].

### 2.4 Outcomes of Interest

The outcomes of interest for this study were encounters in which a foot disease condition was recorded as a problem managed. Foot disease was defined as a condition of the foot or lower leg (i.e., below the knee) that included neuropathy, ischaemia/peripheral artery disease, ulceration, infection or amputation‐related condition [3]. To identify encounters, we selected ICPC–2 PLUS terms for conditions that were primarily related to foot disease problems (Supporting Information S1: Appendix Table A2) [11, 18, 19, 20]. We refer to these collective conditions as foot disease and sub‐categorised these conditions into neuropathy, ischaemia, ulceration, infection or amputation‐related foot disease problems [11, 20].

We also used data from relevant BEACH sub‐studies conducted between 2012 and 2016 to explore encounters at which diabetes was confirmed. In those BEACH sub‐studies, the GP also recorded if the patient had diagnosed type 1 or type 2 diabetes in a sub‐set of 30 patients (from the original 100) [18]. We then linked the encounter data with the sub‐study data for the years 2012–2013 to 2015–2016 to compare the management of foot disease at encounters in which patients had diagnosed diabetes (type 1 or type 2) and those who did not [18].

### 2.5 Statistical Analyses

The BEACH dataset had a single‐stage cluster design with each GP (unit of sampling) having a cluster of 100 encounters (unit of inference). We used ‘survey means’ procedures in SAS version 9.4 (SAS Inc., Cary, NC, USA) to produce 95% confidence intervals (95% CIs) to account for the effect of clustering [18]. As more than one problem could be managed per GP‐patient encounter, foot disease management rates were reported per 1000 GP‐patient encounters. As more than one action could be provided per problem managed within each encounter, management action rates were reported per 100 foot disease problems managed. To further estimate the number of GP foot disease encounters per head of the Australian population, we extrapolated the foot disease management rate to the number of nonreferred Medicare Benefits Schedule GP attendances claimed each year in Australia [18], divided by the annual Australian population [21]. Statistical significance was defined when 95% CIs did not overlap as used in other studies analysing BEACH data [18]. Multivariable logistic regression models were used to examine the variables that were independently associated with GP foot disease encounters. All variables collected were entered into the model and adjusted odds ratios reported for each variable. Multicollinearity was tested using the correlation matrix and only practice location and patient relative socio‐economic level showed any collinearity (0.51). Missing data were removed from all analyses. The proportion of data that was missing was less than 10% for each variable.

## 3 Results

### 3.1 GP Encounter Rates

**FIGURE 1:**
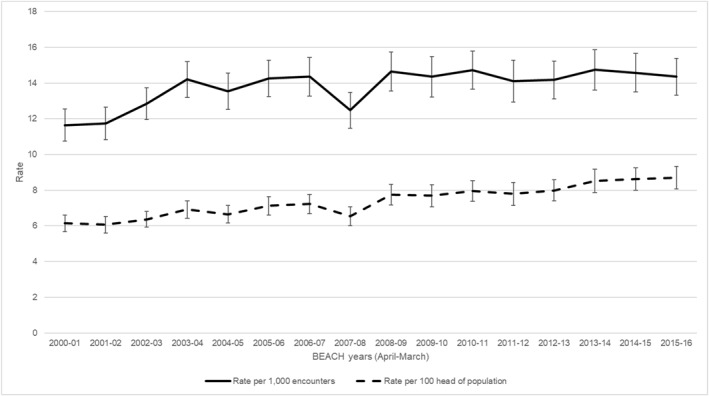
The management rate of foot disease problems by Australian general practitioners between April 2000 and March 2016. Solid line represents problems per 1000 encounters and dashed line represents problems per 100 head of population (error bars represent 95% CIs).

**FIGURE 2:**
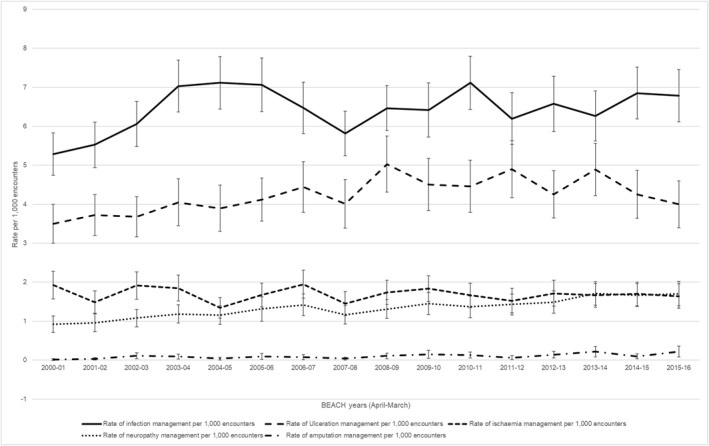
The management rate of different foot disease problem sub‐categories by Australian general practitioners per 1000 encounters between April 2000 and March 2016. Solid line represents infection problems, dashed line represents ulceration problems, square dot line represents ischaemia problems, round dot line represents neuropathy problems and dash dot line represents amputation problems per 1000 encounters.

For the 16‐year period, 15,681 GPs recorded 1,568,100 patient encounters, of which 21,700 involved foot disease management at a rate of 13.8 (95% CI: 13.5–14.1) per 1000 GP encounters, including 6.4 (6.3–6.6) for infection, 4.2 (4.1–4.4) for ulceration, 1.7 (1.6–1.8) for ischaemia, 1.3 (1.3–1.4) for neuropathy and 0.10 (0.08–0.12) for amputation‐related sub‐categories. There was a 24% increase in GP foot disease management rates, from 11.6 (10.8–12.5) per 1000 GP encounters in 2000–2001 to 14.4 (13.3–15.4) in 2015–2016 (Figure 1 and Supporting Information S1: Appendix Table A3), including 28% for infection (5.3 [4.7–5.8]–6.8 [6.1–7.5]), 85% for neuropathy (0.9 [0.7–1.1]–1.7 [1.4–2.0]) and 1100% for amputation‐related sub‐categories (0.02 [0.00–0.04]–0.22 [0.08–0.36]) (Figure 2 and Supporting Information S1: Appendix Table A3). The GP foot disease management rate per head of the Australian population increased by 43% from 6.1 (5.7–6.6) in 2000–2001 to 8.7 (8.1–9.3) per 100 people in 2015–16 (Figure 1 and Supporting Information S1: Appendix Table A3).

For the 4‐year sub‐study period, 1626 GPs recorded 48,689 patient encounters, including 5086 (10.4%) in patients with diabetes and 43,603 (89.6%) without diabetes (Table 1). At the diabetes encounters, 160 (22.2%) involved foot disease management at a rate of 31.5 (26.4–36.6) per 1000 GP encounters with diabetes (Table 2), a 2.4‐fold higher rate than in the nondiabetes encounters, of which 562 involved foot disease management at a rate of 12.9 (11.8–14.0) per 1000 GP encounters without diabetes (Table 3). All foot disease sub‐category rates were significantly higher in patients with diabetes, ranging from 3.2‐fold for ulceration to 1.8‐fold for infection, except amputation (Table 3).

**TABLE 1:** Patient and general practitioner (GP) specific management rate of foot disease per 1000 GP encounters, 2012–2016.

Patient characteristics	Sample size (*n* = 48,689)	Number of problems managed (*n* = 722)	Distribution (%) of problems managed by patient and GP characteristics	Characteristic specific rate of problems per 1000 encounters	Adjusted odds ratios of a problem being managed at encounter (95% CIs)^a^
Sex *(missing)*	(388)	(5)			*p* = 0.0027
Male	19,518	337	47.0	17.3 (15.4–19.2)	1.299 (1.095–1.541)
Female	28,783	380	53.0	13.2 (11.8–14.6)	Reference group
Age *(missing)*	(906)	(5)			*p* < 0.0001
0–24 years	9065	52	7.3	5.7 (4.2–7.3)	Reference group
25–39 years	7152	44	6.1	6.2 (4.2–8.1)	1.022 (0.647–1.614)
40–59 years	12,514	136	19.0	10.9 (9.0–12.7)	1.836 (1.283–2.628)
60–80 years	13,674	292	40.7	21.4 (18.8–23.9)	3.122 (2.216–4.397)
80+ years	5378	193	26.9	35.9 (30.4–41.3)	5.074 (3.506–7.343)
Socioeconomic level *(missing)*	(1010)	(11)			*p* = 0.7263
Disadvantaged	18,626	303	42.6	16.3 (14.4–18.2)	Reference group
Advantaged	29,053	408	57.4	14.0 (12.6–15.5)	0.965 (0.793–1.176)
Healthcare card *(missing)*	(4008)	(54)			*p* = 0.0201
Healthcare card	20,515	423	63.3	20.6 (18.5–22.7)	1.266 (1.038–1.544)
No healthcare card	24,166	245	36.7	10.1 (8.8–11.4)	Reference group
Language background *(missing)*	(4829)	(66)			*p* = 0.0140
Non‐English speaking	3812	36	5.5	9.4 (6.3–12.6)	Reference group
English speaking	40,048	620	94.5	15.5 (14.2–16.8)	1.574 (1.096–2.259)
Indigenous status *(missing)*	(4830)	(65)			*p* = 0.2119
Indigenous	911	7	1.1	7.7 (2.2–13.1)	0.636 (0.312–1.295)
Nonindigenous	42,948	650	98.9	15.1 (13.9–16.4)	Reference group
GP sex *(missing)*	(0)				*p* = 0.9224
Male	27,869	443	61.4	15.9 (14.3–17.5)	0.991 (0.828–1.186)
Female	20,820	279	38.6	13.4 (11.8–15.0)	Reference group
GP age *(missing)*	(270)	(7)			*p* = 0.2967
< 45 years	12,421	174	24.3	14.0 (11.7–16.3)	Reference group
45–54 years	13,466	185	25.9	13.7 (11.6–15.9)	0.858 (0.676–1.089)
55+ years	22,532	356	49.8	15.8 (14.1–17.4)	0.847 (0.683–1.051)
Practice location *(missing)*	(120)	(1)			*p* = 0.8696
Major cities	34,051	484	67.1	14.2 (12.9–15.5)	1.074 (0.788–1.463)
Inner regional	9561	159	22.1	16.6 (13.9–19.4)	1.088 (0.792–1.494)
Outer regional/remote	4957	78	1.8	15.7 (12.1–19.4)	Reference group
Country of graduation *(missing)*	(150)	(2)			*p* = 0.1791
Australian graduate	32,242	503	69.9	15.6 (14.2–17.0)	Reference group
Overseas graduate	16,297	217	30.1	13.3 (11.4–15.2)	0.882 (0.734–1.059)
Diabetes status *(missing)*	(0)				*p* < 0.0001
Diabetes diagnosed	5086	160	22.2	31.5 (26.4–36.6)	1.586 (1.292–1.948)
No diabetes diagnosed	43,603	562	77.8	12.9 (11.8–14.0)	Reference group
Total	48,689		100.0		14.82 (13.69–15.97)

^a^ A multivariable logistic regression model was used to examine all characteristics to identify those independently associated with GP foot disease encounters. All patient and GP characteristics were entered and adjusted for in the model and adjusted odds ratios reported for each characteristic. There were 41,781 encounters included in the model after 6520 were excluded due to missing data.

**TABLE 2:** Patient and general practitioner (GP) specific management rate of foot disease per 1000 GP encounters with diabetes, 2012–2016.

Patient characteristics	Sample size (*n* = 5086)	Number of problems managed (*n* = 160)	Distribution (%) of problems managed by patient and GP characteristics	Characteristic specific rate of problems per 1000 encounters with diabetes	Adjusted odds ratios of a problem being managed at encounter with diabetes (95% CIs)^a^
Sex *(missing)*	(33)	(0)			*p* = 0.0059
Male	2423	92	57.5	38.0 (29.8–46.2)	1.678 (1.161–2.423)
Female	2630	68	42.5	25.9 (19.7–32.0)	Reference group
Age *(missing)*	(61)	(0)			*p* = 0.5550
0–24 years	53	1	0.6	18.9 (0.0–55.5)	Reference group
25–39 years	138	3	1.9	21.7 (0.0–46.1)	0.790 (0.070–8.913)
40–59 years	1086	24	15.0	22.1 (12.4–31.8)	0.946 (0.120–7.449)
60–80 years	2762	91	56.9	32.9 (26.1–39.8)	1.366 (0.179–10.431)
80+ years	986	41	25.6	41.6 (28.1–55.1)	1.590 (0.203–12.478)
Socio‐economic level *(missing)*	(94)	(1)			*p* = 0.8053
Disadvantaged	2399	77	48.4	32.1 (24.5–39.7)	Reference group
Advantaged	2593	82	51.6	31.6 (24.5–38.7)	0.946 (0.607–1.474)
Healthcare card *(missing)*	(286)	(15)			*p* = 0.1174
Healthcare card	3470	115	79.3	33.1 (26.9–39.4)	1.448 (0.911–2.303)
No healthcare card	1330	30	20.7	22.6 (14.1–31.0)	Reference group
Language background *(missing)*	(335)	(16)			*p* = 0.0824
Non‐English speaking	544	10	6.9	18.4 (7.3–29.5)	Reference group
English speaking	4207	134	93.1	31.9 (26.2–37.5)	1.778 (0.929–3.403)
Indigenous status *(missing)*	(340)	(16)			*p* = 0.3755
Indigenous	212	3	2.1	14.2 (0.0–29.8)	0.604 (0.198–1.843)
Nonindigenous	4534	141	97.9	31.1 (25.8–36.4)	Reference group
GP sex *(missing)*	(0)	(0)			*p* = 0.1987
Male	3179	99	61.9	31.1 (24.7–37.6)	0.774 (0.523–1.145)
Female	1907	61	38.1	32.0 (23.6–40.3)	Reference group
GP age *(missing)*	(28)	(1)			*p* = 0.1467
< 45 years	1080	40	25.2	37.0 (24.5–49.6)	Reference group
45–54 years	1434	35	22.0	24.4 (16.5–32.3)	0.608 (0.369–1.001)
55+ years	2544	84	52.8	33.0 (25.6–40.4)	0.790 (0.505–1.235)
Practice location *(missing)*	(11)	(1)			*p* = 0.8031
Major cities	3265	105	66.0	32.2 (25.8–38.5)	1.249 (0.587–2.655)
Inner regional	1141	35	22.0	30.7 (20.2–41.1)	1.099 (0.528–2.290)
Outer regional/remote	669	19	11.9	28.4 (13.3–43.5)	Reference group
Country of graduation *(missing)*	(24)	(1)			*p* = 0.0718
Australian graduate	3333	114	71.7	34.2 (27.7–40.7)	Reference group
Overseas graduate	1729	45	28.3	26.0 (17.8–34.2)	0.689 (0.460–1.034)
Total	5086	160	100.0	31.46 (26.35–36.56)	

^a^ A multivariable logistic regression model was used to examine the characteristics above that were independently associated with GP foot disease encounters. All patient and GP characteristics were entered and adjusted for in the model and adjusted odds ratios reported for each characteristic. There were 4558 encounters included in the model after 495 were removed due to missing data.

**TABLE 3:** General practitioner (GP) management rate of foot disease sub‐categories per 1000 GP encounters, 2012–2016 by diabetes status.

	All		Nondiabetes		Diabetes	
Foot disease sub‐category	*n* = 48,689 encounters	Rate per 1000 encounters (95% CIs)	*n* = 43,603 encounters	Rate per 1000 encounters (95% CIs)	*N* = 5086 encounters	Rate per 1000 encounters (95% CIs)
Infection	322	6.6 (5.9–7.35)	267	6.1 (5.4–6.9)	55	10.8 (7.8–13.8)^a^
Bacterial	149	3.1 (2.6–3.6)	119	2.7 (2.2–3.2)	30	5.9 (3.7–8.1)^a^
Fungal	153	3.1 (2.6–3.7)	136	3.1 (2.6–3.7)	17	3.3 (1.8–4.9)
Unspecified	20	0.4 (0.2–0.6)	12	0.3 (0.1–0.4)	8	1.6 (0.5–2.7)^a^
Ulceration	205	4.2 (3.6–4.9)	149	3.4 (2.8–4.0)	56	11.0 (7.8–14.2)^a^
Ischaemia	110	2.3 (1.8–2.7)	84	1.9 (1.5–2.4)	26	5.1 (3.2–7.1)^a^
Neuropathy	77	1.6 (1.2–1.9)	56	1.3 (0.9–1.6)	21	4.1 (2.3–6.0)^a^
Amputation	8	0.2 (0.1–0.3)	6	0.1 (0.0–0.2)	2	0.4 (0.0–0.9)
Total foot disease	722	14.8 (13.7–16.0)	562	12.9 (11.8–14.0)	160	31.5 (26.4–36.6)^a^

^a^ Statistical significance difference: defined when 95% confidence intervals do not overlap.

### 3.2 Factors Associated

Patient factors independently associated with higher GP encounters for foot disease problems were males, older age (> 40 years), diabetes diagnosis, English‐speaking background and holding a healthcare concession card (all, *p* < 0.05; Table 1). In those with diabetes, only males were independently associated with higher GP encounters for foot disease problems (*p* < 0.05; Table 2).

### 3.3 Management Actions Performed

The most frequent actions used by GPs to manage foot disease problems were medications at a rate of 59.6 (95% CI: 54.8–64.3) per 100 foot disease problems (such as antibiotics 23.5, antifungals 12.6), procedures 30.7 (26.6–34.9; such as wound dressings 25.5) and pathology 24.4 (18.4–30.4; such as full blood count 3.3, fungal scraping/culture 3.2; Table 4). Least frequent actions used by GPs were referrals to other health professionals 10.9 (8.5–13.4; such as vascular surgeons 3.0, podiatrists 1.8), providing counselling/advice/education 9.7 (7.4–12.0) and imaging 4.5 (3.0–6.1; such as ultrasound 2.4, x‐rays 1.0). The only management action that was different by diabetes status was using fewer antifungals in patients with diabetes (6.3 [2.5–10.0] vs. 14.4 [11.3–17.5]).

**TABLE 4:** Management actions used by a general practitioner (GP) per 100 foot disease problems, 2012–2016 by diabetes status.^a^

Management action		All		Non‐diabetes		Diabetes
	*n*	Rate per 100 problems (95% CIs) *n* = 722	*n*	Rate per 100 problems (95% CIs) *n* = 562	*n*	Rate per 100 problems (95% CIs) *n* = 160
Medication	430	59.6 (54.8–64.3)	348	61.9 (56.4–67.4)	82	51.3 (42.0–60.5)
Antibiotics	170	23.5 (20.4–26.7)	133	23.7 (20.0–27.3)	37	23.1 (16.4–29.9)
Cephalexin	85	11.8 (9.4–14.2)	71	12.6 (9.8–15.5)	14	8.8 (4.3–13.2)
Flucloxacillin	23	3.2 (1.8–4.5)	18	3.2 (1.7–4.7)	5	3.1 (0.4–5.8)
Dicloxacillin	14	1.9 (0.8–3.0)	11	2.0 (0.7–3.2)	3	1.9 (0.0–4.0)
Antifungals	91	12.6 (10.0–15.2)	81	14.4 (11.3–17.5)	10	6.3 (2.5–10.0)^b^
Clotrimazole	23	3.2 (1.8–4.5)	22	3.9 (2.2–5.6)	1	0.6 (0.0–1.9)^b^
Terbinafine (oral)	20	2.8 (1.6–4.0)	19	3.4 (1.9–4.9)	1	0.6 (0.0–1.9)^b^
Terbinafine (topical)	20	2.8 (1.6–4.0)	18	3.2 (1.6–4.8)	2	1.3 (0.0–3.0)
Analgesics	26	3.6 (1.8–5.4)	17	3.0 (1.2–4.8)	9	5.6 (1.3–10.0)
Opioid	23	3.2 (1.5–4.9)	14	2.5 (0.9–4.1)	9	5.6 (1.3–10.0)
Nonopioid	3	0.4 (0.0–0.9)	3	0.5 (0.0–1.1)	0	0.0 (0.0–0.0)
Anti‐Parkinson	21	2.9 (1.6–4.2)	16	2.8 (1.4–4.3)	5	3.1 (0.4–5.8)
Pramipexole	16	2.2 (1.2–3.3)	11	2.0 (0.8–3.1)	5	3.1 (0.4–5.8)
Corticosteroid (topical)	20	2.8 (1.5–4.0)	17	3.0 (1.5–4.5)	3	1.9 (0.0–4.0)
Antiepileptic	10	1.4 (0.3–2.5)	7	1.2 (0.3–2.2)	3	1.9 (0.0–5.5)
Procedures	222	30.7 (26.6–34.9)	165	29.4 (24.6–34.2)	57	35.6 (27.4–43.9)
Wound dressing	184	25.5 (22.0–29.0)	133	23.7 (19.7–27.6)	51	31.9 (24.3–39.5)
Pathology	176	24.4 (18.4–30.4)	146	26.0 (19.0–32.9)	30	18.8 (7.1–30.4)
Full blood count	24	3.3 (2.0–4.6)	19	3.4 (1.9–4.9)	5	3.1 (0.4–5.8)
Fungal scraping/culture	23	3.2 (1.8–4.6)	20	3.6 (1.9–5.2)	3	1.9 (0.0–4.0)
Nail scraping/culture	14	1.9 (0.9–3.0)	14	2.5 (1.1–3.9)	0	0.0 (0.0–0.0)
Skin swab/culture	11	1.5 (0.6–2.4)	10	1.8 (0.7–2.9)	1	0.6 (0.0–1.9)
Electrolytes liver function test	10	1.4 (0.5–2.2)	10	1.8 (0.7–2.9)	0	0.0 (0.0–0.0)
C‐reactive protein	9	1.2 (0.4–2.1)	7	1.2 (0.3–2.2)	2	1.3 (0.0–3.0)
Liver function	8	1.1 (0.3–1.9)	6	1.1 (0.2–1.9)	2	1.3 (0.0–3.0)
Electrolytes, urea, creatinine	7	1.0 (0.3–1.7)	5	0.9 (0.1–1.7)	2	1.3 (0.0–3.0)
Referral	79	10.9 (8.5–13.4)	58	10.3 (7.8–12.8)	21	13.1 (7.2–19.1)
Vascular surgeon	22	3.0 (1.8–4.3)	16	2.8 (1.5–4.2)	6	3.8 (0.8–6.7)
Podiatrist	13	1.8 (0.8–2.8)	10	1.8 (0.7–2.9)	3	1.9 (0.0–4.0)
Physiotherapist	4	0.6 (0.0–1.1)	3	0.5 (0.0–1.1)	1	0.6 (0.0–1.8)
Counselling/advice/education	70	9.7 (7.4–12.0)	58	10.3 (7.7–13.0)	12	7.5 (3.4–11.6)
Imaging	33	4.5 (3.0–6.1)	26	4.6 (2.8–6.4)	7	4.4 (1.2–7.6)
Ultrasound	17	2.4 (1.2–3.5)	12	2.1 (0.9–3.3)	5	3.1 (0.4–5.8)
X‐ray	7	1.0 (0.3–1.7)	5	0.9 (0.1–1.7)	2	1.3 (0.0–3.0)
Computerised tomography scan	7	1.0 (0.3–1.7)	7	1.2 (0.3–2.2)	0	0.0 (0.0–0.0)

^a^ Note: Only actions with overall rates > 1.0 are reported.

^b^ Statistical significance difference: defined when 95% confidence intervals do not overlap.

## 4 Discussion

Several important novel findings were identified in this study. First, foot disease conditions were managed more than once per 100 GP encounters, which can be extrapolated to nearly one GP foot disease encounter per 10 Australians per year, with most being infection or ulceration. Second, GP foot disease management rates increased by 24% over a recent 16‐year period in Australia, driven mostly by increases in infection and neuropathy. Third, GP foot disease management rates were much higher within patients with diabetes, but GP foot disease management encounter numbers were much higher in patients without diabetes. Fourth, patient factors associated with higher GP foot disease management encounters were diabetes diagnosis, male sex, older age, English‐speaking background and healthcare card holders. Fifth, in people with diabetes, only male sex was associated with higher foot disease management. Finally, regardless of diabetes, the most frequent GP management actions for foot disease were using medications, procedures and pathology, and the least frequent were referrals, counselling and imaging.

We found foot disease conditions were managed at a rate of 1.4 per 100 GP encounters, and this can be extrapolated to 8.7 GP foot disease encounters per 100 Australian people per year, with rates increasing significantly over time. Interpreted with similar papers, this suggests the vast majority of foot‐related conditions managed by GPs are either foot disease or foot pain [11, 12, 13, 14]. Also as other foot‐related conditions had stable trends in the same dataset and period [12, 13, 14, 20], the 24% increase we found in GP foot disease encounter rates suggests foot disease was the major driver of the overall 18% increase in all GP foot‐related condition encounters [11]. Furthermore, when compared to all other health problems managed by GPs in the same nationally representative dataset and period, our findings also suggest foot disease was the 21st most frequently managed health problem by GPs and increasing (1.2 in 2007 to 1.4 per 100 GP encounters in 2016) [17, 18]. In comparison, this was lower than hypertension (9.6–7.5; 1st) and diabetes (3.6–4.0; 5th), but higher than ischaemic heart disease (1.3–0.9; 36th) and chronic obstructive pulmonary disease (0.8–0.9; 38th) [17, 18]. Extrapolating our findings to the 143 million total GP encounters occurring across Australia in 2015–2016 suggests 2.1 million GP consultations involved managing foot disease in that year [18]. Thus, strategies are needed to increase policy‐maker awareness of the comparatively large and growing burden of foot disease in the community and the importance of evidence‐based primary care management by GPs to potentially reduce this burden in the future [2, 15, 22, 23, 24, 25].

Although GP foot disease management rates increased by 24% across the 16‐year period, the most significant changes occurred in 2003–2004 (increased), 2007–2008 (decreased) and 2008–2009 (increased again). Whilst significant changes in those years were not as evident in other BEACH studies [11, 13, 14, 17, 20], we note significant Australian and State Government policy changes that potentially impacted foot‐related primary care around those specific years [26, 27, 28, 29, 30]. For example, the Australian Government introduced new Medicare (Australia's universal primary healthcare insurance scheme) items in 2003–2004 to fund chronic disease management by allied health professionals [26]. These reforms led to a 5‐fold increase in referrals to podiatrists by GPs after 2003–2004, with most of those referrals (20%–25%) being for diabetes‐related foot problems [26]. Conversely, GP utilisation of these same Medicare items declined in 2007–2008 due to growing administrative burdens [27]. This decline precipitated further Australian Government Medicare policy reforms in 2009–2010 to reduce the administrative burdens on GPs [27]. Further, some Australian State Governments began to increase public health funding to increase access to multi‐disciplinary foot disease teams for people with foot disease around 2007–2008 [28, 29, 30]. Thus, we hypothesise that these government policy changes may have influenced GP foot disease management rate changes, but further research investigating the impact of such policy changes on GP foot disease management practices is needed.

We also found much higher rates (2–3 fold) of GP foot disease management in populations with diabetes compared to those without diabetes. Considering previous studies have also found foot disease hospitalisation and amputation rates are up to 41‐fold higher in people with diabetes, this is unsurprising [1, 4, 5, 6]. However, although rates were much higher in those with diabetes, the numbers were much higher in those without diabetes (78% of all encounters). Previous studies have also found that foot disease disproportionately affects people with diabetes, but overall numbers of foot disease‐related consultations and hospitalisations were higher in people without diabetes [4, 5, 6]. Thus, these findings confirm that people with diabetes should remain a focus of foot disease management, but there are also substantial numbers of people without diabetes who need foot disease management.

GP foot disease encounters were not only associated with diabetes but also older ages, males, English‐speaking backgrounds and healthcare card holders. Older age was unsurprising considering this was found in most other foot‐related conditions [11, 12, 13, 14]. However, interestingly, there was no detectable association with age in patients with diabetes [1, 4]. This may be due to lack of power in our diabetes sub‐group, but does align with smaller studies suggesting primary care encounters managing people with diabetes‐related foot disease were associated with younger age or not associated with age at all [16, 31]. Furthermore, this aligns with recent studies reporting younger age is increasingly associated with poorer diabetes‐related foot disease outcomes and that people with younger‐onset type 2 diabetes may be at higher risk [1, 4, 7, 8, 28]. We also found male sex was associated with foot disease, which has been consistently found in people with diabetes [1, 4, 7, 8, 28], but this seems to suggest this relationship also occurs in people without diabetes. Thus, with females found to be associated with foot pain in similar nationally representative studies [11, 12, 13], this finding suggests males are more likely to present to GPs for foot disease and females for foot pain.

The association with healthcare card holders was also unsurprising, as these concession cards are designed to lower costs and incentivise access to GPs in Australia [11, 14, 26]. The association with English‐speaking backgrounds was also found in similar studies and we also hypothesise that English‐speaking people may find GPs easier to access in predominantly English‐speaking healthcare systems [11, 14, 26]. Finally, we found that Australian GP graduates trended towards providing more foot disease encounters in those with diabetes. This suggests GPs trained in Australia may have greater awareness of managing diabetes‐related foot disease, and perhaps strategies to increase awareness in overseas‐trained GPs should be targeted [11, 15, 23].

The most frequent GP actions to manage foot disease were to use medications (60 times per 100 foot disease problems managed), procedures (31 per 100) and pathology (24 per 100). The high rate of medication use (mainly anti‐infectives) was unsurprising considering infections made up the largest proportion (46%) of GP foot disease encounters and was slightly lower than the average rate of medication use for all health problems (66 per 100 problems) [17], but higher than the rate for all foot‐related problems (46 per 100) [11]. Similarly, the substantial rates of procedures used (mainly wound dressings) was also unsurprising considering ulcers made up the second largest proportion (30%) of GP foot disease encounters, and again the rate fell between the rate of procedure use for all health problems (36 per 100) and for all foot‐related problems (24 per 100) [11, 17]. Furthermore, pathology use also fell between that for all health problems (31 per 100) and all foot‐related problems (19 per 100) [11, 17].

Perhaps most surprising were the management actions used least, including referrals (11 times per 100 foot disease problems managed), counselling (10 per 100) and imaging (5 per 100). Interestingly, the referral rate was lower than that for all foot‐related problems (15 per 100), but similar to all health problems (10 per 100) [11, 17]. Considering guidelines strongly recommend referrals for all people with diabetes‐related foot disease to multi‐disciplinary foot teams, the referral rate of 13 per 100 diabetes‐related foot disease problems to any healthcare professional still seems very low [22, 30], even considering a reported 5‐fold increase in GP referrals to podiatrists during the period [26]. However, this could have also been impacted by the BEACH cross‐sectional study design which was not designed to capture referrals over time for individual patients. Further research is needed, and strategies developed, to optimise referrals to multi‐disciplinary foot teams in future [16, 31].

Counselling and imaging rates also seemed disproportionately low (10 and 5 times, respectively, per 100 foot disease problems managed) with the equivalent rates higher for all health problems (25 and 7) and for all foot‐related problems (15 and 19) [11, 17]. Considering guidelines recommend all people with diabetes‐related foot disease receive counselling/education and most also require imaging for infection and ischaemia in particular, this seems a lost opportunity [22, 30]. These disproportionately low rates of referrals, counselling and imaging, fit with previous qualitative studies that identified there are multiple barriers for GPs delivering foot disease management, including a historical lack of guidelines, limited multi‐disciplinary foot teams and geographical remoteness [16, 23]. Identified enablers though have included increased training, guideline‐recommended clinical pathways, service incentives and improved access to multi‐disciplinary foot team [23, 24, 25]. Thus, we recommend that strategies incentivising GPs to manage, counsel and refer in adherence with guideline‐recommended care are also implemented to prevent more foot disease‐related hospitalisations [15, 22, 23, 24, 25, 30].

### 4.1 Limitations

This study should be read cognisant of several limitations. First, although this study provides the most extensive insight into foot disease management by GPs, it used cross‐sectional data and was unable to identify how individual patients were managed over time [11, 17]. Thus, the large gap between real‐world GP management actions found in this study and guideline‐recommended management may be smaller than these findings suggest. Second, although we analysed data over 16 years, the most recent was from 2016 and GP management may have improved. However, although there has been some focus on foot disease management in secondary and tertiary care with associated improvements in outcomes for people with foot disease in some Australia regions [28, 29, 30], and potentially large increases in referrals to podiatrists by GPs for diabetes‐related foot problems [26], to our knowledge, there had been no such focus on evidence‐based foot disease management by GPs in Australian primary care. Furthermore, considering the 2011 Australian evidence‐based guidelines for diabetes‐related foot disease were only recently updated in 2023 [30], we expect any improvements in foot disease management by GPs was minimal during the period. However, promisingly our findings do suggest government policy changes can influence GP foot disease management. Third, while we defined foot disease according to international standards and identified foot disease using well‐structured ICPC‐2 PLUS codes [3, 18, 19], our data still relied on GPs accurately recording and coders accurately coding foot disease problems. However, previous studies have reported an under‐estimate of foot disease problems using similar methods in other datasets and this may mean our findings could also be underestimates as well [11, 12, 13, 14]. Fourth, we used a conservative definition of statistical significance as per other BEACH studies, so as to not overstate our findings where we made multiple comparisons [18]. Thus, our findings could be prone to false negative conclusions, but conversely our findings are likely robust. Finally, these findings are from Australian GPs and may not be generalisable to GPs from other countries; however, the Australian BEACH data have been reported to be comparable to both United Kingdom and United States primary care data [12, 32].

## 5 Conclusions

GP consultations for managing foot disease were more common and increasing more rapidly than many other well‐known conditions, such as ischaemic heart disease and chronic obstructive pulmonary disease. Patient characteristics associated with higher GP foot disease management rates not only included those with diabetes but also being male, older ages, English‐speaking backgrounds and healthcare card holders. Regardless of diabetes status, GPs mostly used medications and procedures to manage foot disease but rarely counselled or referred. With this being the first study to explore real‐world foot disease management by GPs in nationally representative primary care services, these findings should considerably improve our understandings of the gap between real‐world practice and guideline‐recommended foot disease management in people with and without diabetes. These findings should enable governments and policymakers to develop strategies to close this gap and reduce large national foot disease burdens in future.

## Author Contributions

**Peter A. Lazzarini:** conceptualization, methodology, writing – original draft, writing – review and editing. **Hylton B. Menz:** conceptualization, methodology, writing – review and editing. **Cylie M. Williams:** conceptualization, methodology, writing – review and editing. **Julie Gordon:** conceptualization, methodology, writing – review and editing. **Susanna Cramb:** conceptualization, methodology, writing – review and editing. **Christopher Harrison:** conceptualization, methodology, data curation, formal analysis, writing – review and editing, funding acquisition.

## Conflicts of Interest

P.A.L. and C.W.M. are members of the editorial board, and H.M.B. past editor in chief, of the Journal of Foot and Ankle Research. Otherwise, the authors declare that they have no relevant competing interests.

## Supporting information

Supporting Information S1

## Data Availability

The data that support the findings of this study are available from the BEACH study (https://www.sydney.edu.au/medicine‐health/our‐research/research‐centres/bettering‐the‐evaluation‐and‐care‐of‐health.html), the University of Sydney, but restrictions apply to the availability of these data, which were used under licence for the current study, and so are not publicly available. Data are however available from the authors upon reasonable request and with permission of the BEACH study, the University of Sydney.
