# Supplementary material for: Foot Disease Management by General Practitioners in People With and Without Diabetes: An Analysis of Nationally Representative Primary Care Data in Australia
Source: J Foot Ankle Res. 2025 Aug 28;18(3):e70066. doi: 10.1002/jfa2.70066 (PMC12394062; doi:10.1002/jfa2.70066)
Supplement: Supplementary file 1 — Supporting Information S1 [file JFA2-18-e70066-s001.docx]

**Appendix material**

**Table A1:** STROBE Statement—checklist of items that should be included in reports of observational studies

|  | Item No | Recommendation | Page number | |  |
| --- | --- | --- | --- | --- | --- |
| **Title and abstract** | 1 | (*a*) Indicate the study’s design with a commonly used term in the title or the abstract | 2 | |  |
|  |  | (*b*) Provide in the abstract an informative and balanced summary of what was done and what was found | 2 | |  |
| Introduction | | |  | |  |
| Background/rationale | 2 | Explain the scientific background and rationale for the investigation being reported | 3 | |  |
| Objectives | 3 | State specific objectives, including any prespecified hypotheses | 3 | |  |
| Methods | | |  | |  |
| Study design | 4 | Present key elements of study design early in the paper | 4 | |  |
| Setting | 5 | Describe the setting, locations, and relevant dates, including periods of recruitment, exposure, follow-up, and data collection | 4 | |  |
| Participants | 6 | (*a*) *Cohort study*—Give the eligibility criteria, and the sources and methods of selection of participants. Describe methods of follow-up  *Case-control study*—Give the eligibility criteria, and the sources and methods of case ascertainment and control selection. Give the rationale for the choice of cases and controls  *Cross-sectional study*—Give the eligibility criteria, and the sources and methods of selection of participants | 4 | |  |
|  |  | (*b*) *Cohort study*—For matched studies, give matching criteria and number of exposed and unexposed  *Case-control study*—For matched studies, give matching criteria and the number of controls per case | NA | |  |
| Variables | 7 | Clearly define all outcomes, exposures, predictors, potential confounders, and effect modifiers. Give diagnostic criteria, if applicable | 4-6 | |  |
| Data sources/ measurement | 8* | For each variable of interest, give sources of data and details of methods of assessment (measurement). Describe comparability of assessment methods if there is more than one group | 4-6 | |  |
| Bias | 9 | Describe any efforts to address potential sources of bias | 6 | |  |
| Study size | 10 | Explain how the study size was arrived at | 4 | |  |
| Quantitative variables | 11 | Explain how quantitative variables were handled in the analyses. If applicable, describe which groupings were chosen and why | 4-6 | |  |
| Statistical methods | 12 | (*a*) Describe all statistical methods, including those used to control for confounding | | 6 | |
|  |  | (*b*) Describe any methods used to examine subgroups and interactions | | 6 | |
|  |  | (*c*) Explain how missing data were addressed | | 6 | |
|  |  | (*d*) *Cohort study*—If applicable, explain how loss to follow-up was addressed  *Case-control study*—If applicable, explain how matching of cases and controls was addressed  *Cross-sectional study*—If applicable, describe analytical methods taking account of sampling strategy | | 6 | |
|  |  | (*e*) Describe any sensitivity analyses | | 6 | |
| Results | | | | |  |
| Participants | 13* | (a) Report numbers of individuals at each stage of study—eg numbers potentially eligible, examined for eligibility, confirmed eligible, included in the study, completing follow-up, and analysed | | | 7 |
|  |  | (b) Give reasons for non-participation at each stage | | | 7 |
|  |  | (c) Consider use of a flow diagram | | | NA |
| Descriptive data | 14* | (a) Give characteristics of study participants (eg demographic, clinical, social) and information on exposures and potential confounders | | | 7-8 & Tables 1&2 |
|  |  | (b) Indicate number of participants with missing data for each variable of interest | | | Tables 1&2 |
|  |  | (c) *Cohort study*—Summarise follow-up time (eg, average and total amount) | | | NA |
| Outcome data | 15* | *Cohort study*—Report numbers of outcome events or summary measures over time | | | NA |
|  |  | *Case-control study—*Report numbers in each exposure category, or summary measures of exposure | | | NA |
|  |  | *Cross-sectional study—*Report numbers of outcome events or summary measures | | | 7-8 |
| Main results | 16 | (*a*) Give unadjusted estimates and, if applicable, confounder-adjusted estimates and their precision (eg, 95% confidence interval). Make clear which confounders were adjusted for and why they were included | | | 7-8 & Tables 1&2 |
|  |  | (*b*) Report category boundaries when continuous variables were categorized | | | Tables 1&2 |
|  |  | (*c*) If relevant, consider translating estimates of relative risk into absolute risk for a meaningful time period | | | NA |
| Other analyses | 17 | Report other analyses done—eg analyses of subgroups and interactions, and sensitivity analyses | | | 7-8 |
| Discussion | | | | |  |
| Key results | 18 | Summarise key results with reference to study objectives | | | 8 |
| Limitations | 19 | Discuss limitations of the study, taking into account sources of potential bias or imprecision. Discuss both direction and magnitude of any potential bias | | | 12-13 |
| Interpretation | 20 | Give a cautious overall interpretation of results considering objectives, limitations, multiplicity of analyses, results from similar studies, and other relevant evidence | | | 9-12 |
| Generalisability | 21 | Discuss the generalisability (external validity) of the study results | | | 9-12 |
| Other information | | | | |  |
| Funding | 22 | Give the source of funding and the role of the funders for the present study and, if applicable, for the original study on which the present article is based | | | 14 |

**Table A2:** ICPC2-Plus codes related to foot disease conditions

| **ICPC-2 Plus code** | **Label** | **Sub-category** |
| --- | --- | --- |
| K92001 | Disease;Buergers | Ischaemia |
| K92002 | Disease;Raynauds | Ischaemia |
| K92003 | Disease;peripheral vascular | Ischaemia |
| K92004 | Gangrene | Ischaemia |
| K92006 | Ischaemia;limb (gangrene) | Ischaemia |
| K92010 | Raynauds phenomenon | Ischaemia |
| K92016 | Vasospasm;peripheral | Ischaemia |
| K92017 | Claudication;intermittent | Ischaemia |
| K92031 | Disease;small vessel | Ischaemia |
| L52009 | Amputation;non-traumatic | Amputation |
| L52012 | Amputation;below knee | Amputation |
| L52013 | Amputation;above knee | Amputation |
| L52016 | Amputation;foot | Amputation |
| L52019 | Amputation;toe(s) | Amputation |
| L99105 | Arthropathy;Charcot | Neuropathy |
| N04001 | Restless legs syndrome | Neuropathy |
| N05001 | Burning;sensation;extremities | Neuropathy |
| N05005 | Tingling;feet/toes | Neuropathy |
| N05006 | Paraesthesia | Neuropathy |
| N06022 | Numbness;toe(s) | Neuropathy |
| N06023 | Numbness;foot | Neuropathy |
| N06024 | Numbness;leg | Neuropathy |
| N18002 | Footdrop | Neuropathy |
| N94012 | Neuropathy; diabetic | Neuropathy |
| N94016 | Mononeuritis;legs | Neuropathy |
| N94018 | Neuritis;peripheral | Neuropathy |
| S09005 | Infection;toe(s) | Infection – Unspecified |
| S09010 | Abscess;toe(s) | Infection – Bacterial |
| S09011 | Cellulitis;toe(s) | Infection – Bacterial |
| S11019 | Infection;ingrown toenail | Infection – Bacterial |
| S74001 | Athletes foot | Infection – Fungal |
| S74004 | Infection;fungus;nail(s) | Infection – Fungal |
| S74005 | Infection;fungus;skin | Infection – Fungal |
| S74006 | Mycosis;skin | Infection – Fungal |
| S74009 | Tinea | Infection – Fungal |
| S74015 | Onychomycosis | Infection – Fungal |
| S74018 | Tinea pedis | Infection – Fungal |
| S74025 | Mycetoma | Infection – Unspecified |
| S76009 | Pitted keratolysis | Infection – Bacterial |
| S76015 | Cellulitis;leg | Infection – Bacterial |
| S76016 | Cellulitis;foot/feet | Infection – Bacterial |
| S97004 | Ulcer;varicose | Ulceration |
| S97008 | Ulcer;leg | Ulceration |
| S97012 | Ulcer;foot | Ulceration |
| S97013 | Ulcer;diabetic | Ulceration |
| S97014 | Ulcer;venous | Ulceration |
| S97016 | Ulcer;toe(s) | Ulceration |

ICPC-2: International Classification of Primary Care, second edition

**Table A3:** The management rate of foot disease problems by Australian general practitioners (GPs) between April 2000 and March 2016, per 1,000 encounters (95% Confidence Intervals) unless otherwise stated^#^

| Foot disease problem | 2000-01 | 2001-02 | 2002-03 | 2003-04 | 2004-05 | 2005-06 | 2006-07 | 2007-08 | 2008-09 | 2009-10 | 2010-11 | 2011-12 | 2012-13 | 2013-14 | 2014-15 | 2015-16 |
| --- | --- | --- | --- | --- | --- | --- | --- | --- | --- | --- | --- | --- | --- | --- | --- | --- |
| Total (foot disease) | 11.6  (10.8-  12.5) | 11.7  (10.8-  12.6) | 12.8  (12.0-  13.7) | 14.2  (13.2-  15.2)* | 13.5  (12.5-  14.6)* | 14.3  (13.2-  15.3)* | 14.4  (13.3-  15.4)* | 12.5  (11.5-  13.5) | 14.7  (13.6-  15.7)* | 14.4 (13.2-  15.5)* | 14.7  (13.7-  15.8)* | 14.1  (12.9-  15.3)* | 14.2  (13.1-  15.2)* | 14.7  (13.6-  15.9) | 14.6  (13.5-  15.7)* | 14.4  (13.3-  15.4)* |
| Infection | 5.3  (4.7-  5.8) | 5.5  (4.9-  6.1) | 6.1  (5.5-  6.6) | 7.0  (6.4-  7.7)* | 7.1  (6.4-  7.8)* | 7.1  (6.4-  7.7)* | 6.5  (5.8-  7.1)* | 5.8  (5.2-  6.4) | 6.5  (5.9-  7.0)* | 6.4  (5.7-  7.1) | 7.1  (6.4-  7.8)* | 6.2  (5.5-  6.9) | 6.6  (5.9-  7.3)* | 6.3  (5.6-  6.9) | 6.9  (6.2-  7.5)* | 6.8  (6.1-  7.5)* |
| Ulceration | 3.5  (3.0-  4.0) | 3.7  (3.2-  4.2) | 3.7  (3.2-  4.2) | 4.1  (3.5-  4.7) | 3.9  (3.3-  4.5) | 4.1  (3.6-  4.7) | 4.4  (3.8-  5.1) | 4.1  (3.4-  4.6) | 5.0  (4.3-  5.7)* | 4.5  (3.8-  5.2) | 4.5  (3.8-  5.1) | 4.9  (4.2-  5.6)* | 4.3  (3.6-  4.9) | 4.9  (4.2-  5.6)* | 4.3  (3.6-  4.9) | 4.0  (3.4-  4.6) |
| Ischaemia | 1.9  (1.6-  2.3) | 1.5  (1.2-  1.8) | 1.9  (1.6-  2.3) | 1.9  (1.5-  2.2) | 1.3  (1.1-  1.6) | 1.7  (1.4-  2.0) | 1.9  (1.6-  2.3) | 1.5  (1.1-  1.8) | 1.7  (1.4-  2.0) | 1.8  (1.5-  2.2) | 1.7  (1.4-  2.0) | 1.5  (1.2-  1.8) | 1.7  (1.4-  2.0) | 1.7  (1.4-  2.0) | 1.7  (1.4-  2.0) | 1.6  (1.3-  2.0) |
| Neuropathy | 0.9  (0.7-  1.1) | 1.0  (0.7-  1.2) | 1.1  (0.9-  1.3) | 1.2  (1.0-  1.4) | 1.2  (0.9-  1.4) | 1.3  (1.0-  1.6) | 1.4  (1.2-  1.7)* | 1.2  (0.9-  1.4) | 1.3  (1.1-  1.6)* | 1.5  (1.2-  1.7)* | 1.4  (1.1-  1.6)* | 1.4  (1.2-  1.7)* | 1.5  (1.2-  1.8)* | 1.7  (1.4-  2.0)* | 1.7  (1.4-  2.0)* | 1.7  (1.4-  2.0)* |
| Amputation | 0.02  (0.00-  0.04) | 0.03 (0.00-0.06) | 0.11 (0.03-0.19) | 0.09 (0.03-0.16) | 0.04 (0.00-0.08) | 0.10 (0.02-0.14) | 0.08 (0.01-0.14) | 0.04 (0.00-0.07) | 0.11 (0.04-0.18)* | 0.15 (0.05-0.25)* | 0.13 (0.05-0.21)* | 0.06 (0.01-0.11) | 0.14 (0.06-0.23)* | 0.22 (0.08-0.35)* | 0.10 (0.04-0.16)* | 0.22  (0.08-  0.36)* |
| Total (foot disease)  per 100 people^#^ | 6.1  (5.7-  6.6) | 6.1  (5.6-  6.5) | 6.4  (5.9-  6.8) | 6.9  (6.4-  7.4) | 6.7  (6.2-  7.2) | 7.1  (6.6-  7.6)* | 7.2  (6.7-  7.8)* | 6.5  (6.0-  7.1) | 7.8  (7.2-  8.3)* | 7.7  (7.1-  8.3)* | 7.9  (7.4-  8.5)* | 7.8  (7.2-  8.4)* | 8.0  (7.4-  8.6)* | 8.5  (7.9-  9.2)* | 8.6  (8.0-  9.3)* | 8.7  (8.1-  9.3)* |

^#^ GP foot disease encounters by head of Australian population; ^*^Statistical significance was defined when 95% Confidence Intervals did not overlap (from in this case the first year in 2000-01)
